# The ecology and adaptive evolution of influenza A interspecies transmission

**DOI:** 10.1111/irv.12412

**Published:** 2016-08-08

**Authors:** Udayan Joseph, Yvonne C. F. Su, Dhanasekaran Vijaykrishna, Gavin J. D. Smith

**Affiliations:** ^1^ Duke‐NUS Medical SchoolSingapore; ^2^ Department of PathologySingapore General HospitalSingapore; ^3^ Duke Global Health InstituteDuke UniversityDurhamNCUSA

**Keywords:** adaptation, pandemic, zoonotic

## Abstract

Since 2013, there have been several alarming influenza‐related events; the spread of highly pathogenic avian influenza H5 viruses into North America, the detection of H10N8 and H5N6 zoonotic infections, the ongoing H7N9 infections in China and the continued zoonosis of H5N1 viruses in parts of Asia and the Middle East. The risk of a new influenza pandemic increases with the repeated interspecies transmission events that facilitate reassortment between animal influenza strains; thus, it is of utmost importance to understand the factors involved that promote or become a barrier to cross‐species transmission of Influenza A viruses (IAVs). Here, we provide an overview of the ecology and evolutionary adaptations of IAVs, with a focus on a review of the molecular factors that enable interspecies transmission of the various virus gene segments.

## 1 Introduction

**Figure 1:**
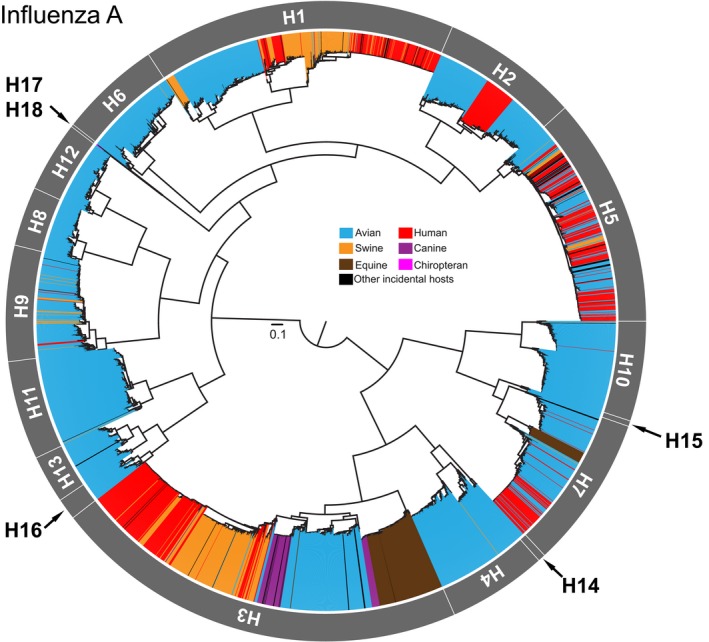
Diversity and host distribution of influenza A viruses (IAVs). Maximum‐likelihood (ML) estimation of the haemagglutinin (HA) gene sequences of all subtypes of IAVs downloaded from the NCBI GenBank database. Overall data set randomly subsampled to include 200 isolates per subtype per host for the tree reconstruction. External branches of tree coloured by the major host groups from which the virus has been isolated from: avian (blue), canine (purple), chiropteran (pink), equine (brown), human (red), swine (amber) and other incidental hosts (black). Scale for branch length represents number of nucleotide substitution per site (subs/site) in the HA alignment

Influenza A virus (IAV) has caused significant morbidity and mortality globally in humans, with an estimated 14 pandemics that have occurred since the 1500s.^1^ Wild aquatic birds are well known to be the natural reservoirs for IAV subtypes harbouring H1–H16 subtypes,^2^, ^3^, ^4^ with the exception of H17 and H18 subtypes that were recently discovered in bats.^5^, ^6^ The phylogenetic relationships of all IAV subtypes are displayed in Fig. 1. In addition to its natural reservoir species, influenza viruses infect a wide range of hosts including canids, equids, humans and swine.^2^ IAVs’ ability to generate novel gene constellations through reassortment between subtypes poses a risk for immune escape in these new hosts.^7^ Furthermore, IAV undergoes rapid genetic and antigenic evolution, which makes vaccination control difficult in humans and other domestic species.

In addition to human pandemics that have emerged from avian and swine hosts, there are also repeated spillover events from domesticated animals, primarily poultry and swine, that pose a significant threat to human health.^8^, ^9^, ^10^, ^11^, ^12^, ^13^, ^14^ Direct transmission of IAV from a wild avian source to humans is rare, as there has only been a single report of laboratory‐confirmed human infection with H5N1 contracted through close contact with dead and infected wild swan in Azerbaijan.^15^ However, there is serological evidence of H5N1 infection among Alaskan hunters who handled dead wild avian species,^16^ indicating that exposure to IAVs from wild birds through close contact can potentially cause infection. More notably, viral genes that are similar to the 1918‐like H1N1 avian virus were recently detected in the influenza gene pools of wild birds, raising the potential for the re‐emergence of a 1918‐like pandemic virus.^17^ Furthermore, due to increasing human encroachment of wildlife habitats, the potential of a wild‐source threat becomes more relevant, as is seen with the emergence of other pathogens such as human immunodeficiency virus (HIV), severe acute respiratory syndrome coronavirus and the more recent Zaire‐variant Ebola virus in Western Africa.^18^, ^19^, ^20^, ^21^

**Figure 2:**
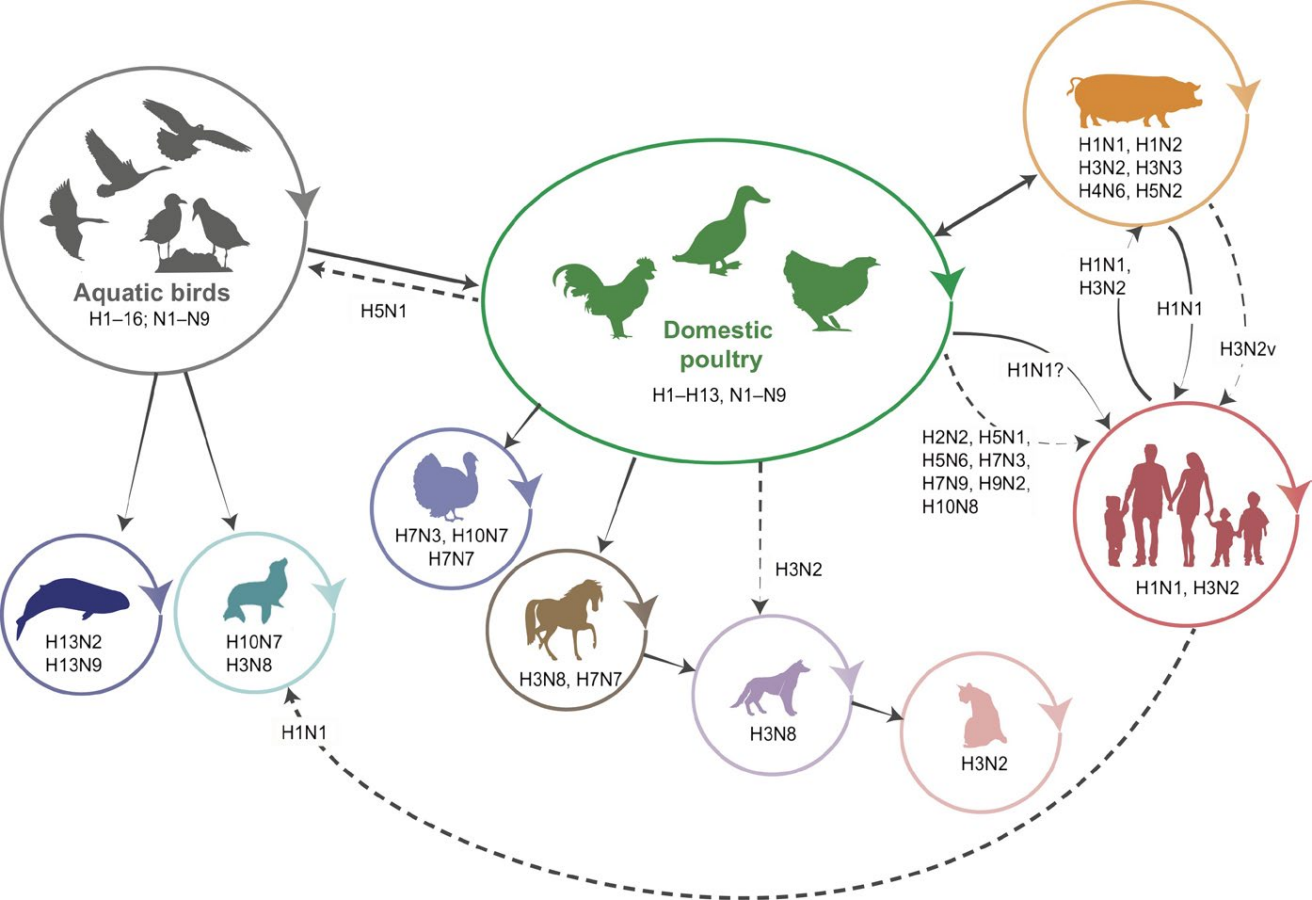
Significant interspecies transmission of influenza A viruses (IAVs). Representative diagram of interspecies transmission events of IAVs and the subtypes involved in these events. Solid arrows represent direct transmission events that have since been established in the host species. Dashed arrows represent sporadic or limited infection of subtypes where sustained transmission in the new host has not been detected

In this review, we discuss the current knowledge of ecological and molecular determinants responsible for interspecies transmission of IAV, with specific focus on avian‐derived influenza subtypes involved in zoonotic and epizootic transmission to other hosts (see Fig. 2).

## 2 Role of wild birds in IAV ecology

Wild birds of the orders Anseriformes (such as ducks and geese) and Charadriiformes (such as shorebirds and gulls) are the primary reservoir of avian influenza viruses.^2^ The maintenance of this diverse pool of viruses globally is in part due to the migratory nature of the bird species, a mechanism in which the viruses are shed in bird faeces and later acquired by other birds that share the same habitat along migratory flyways.^2^, ^22^, ^23^, ^24^ Transmission of IAV in wild bird populations is dependent on several factors including virus shedding, virus stability in the environment and the degree of close contact/mixing with other host species.^3^, ^25^

Virus shedding by bird species in the environment mainly occurs in fresh water, from where viable viruses of most known subtypes currently circulating in wild birds have been isolated.^24^ The persistence of infectivity of IAV shed by aquatic birds is dependent on the temperature and pH of the freshwater, where it has been found that shed viruses can remain infectious for 4 days in temperatures of 28°C and >30 days in temperatures below 0°C, with greatest viral recovery noted at a slightly basic pH (7.4–8.2) and temperatures lower than 17°C.^26^, ^27^, ^28^, ^29^ Prolonged virus shedding, which can be attributed to the development of the avian immune system, facilitates the efficient transmissions of IAVs in their natural hosts.^27^ In the avian cellular immune response, the maturation of T lymphocytes occurs in the thymus glands that are distributed around the neck and inside the thoracic cavity of most birds. Unlike humans, these thymus glands only fully develop in late juvenile birds and persist throughout adult life.^30^ In contrast, the plasmacytoid B cells essential for antibody‐mediated response mature in a specialized organ called the bursa of Fabricius (BF), located in the cloaca, but the BF involutes or disappears early in the juvenile bird stage, that is at the onset of feather growth.^27^, ^30^ The transition from the loss of the BF to the full functionality of the thymus results in a period of a weakened immunity in these avian species; thus migrating juvenile birds with a high viral load become an infection source of different pathogens.^27^, ^31^

The stability of the haemagglutinin (HA) protein, both in the intracellular and extracellular environment, is a key determinant of the influenza virus stability and infectivity in the environment.^32^ Stability of the HA in low intracellular pH of the early endosome is essential for the intracellular entry of the virus, after which point a threshold pH is reached in the late endosome causing the fusion of the protein to the lipid membrane, enabling the viral genome to escape the late endosome and successfully infect the cell.^7^ Conversely, the virus must also maintain its stability in variable extracellular pH environments in its transmission between hosts. Thus, it has been observed that the pH stability of IAV subtypes evolves differently depending on host type and the route of transmission.^33^ As most transmission of avian IAV in aquatic birds is through the oral–faecal route and the replication occurs mostly in the GI tract and cloaca of the birds, these viruses have evolved for stability at higher extracellular pH to accommodate these sites of replication and also maintain viability in the aquatic environment.^33^, ^34^, ^35^, ^36^

More recently, the incorporation of environmental durability data into a phylogenetic study of avian IAV demonstrated that environmental transmission is correlated with the increase in virus genetic diversity in short‐lived avian hosts, suggesting an environmental reservoir can act as a source of annual outbreaks.^37^

## 3 Role of domestic birds in IAV ecology and interspecies transmission to humans and other mammals

AIV can cause low pathogenic infections (LPAI) in domestic birds with an asymptomatic or mild disease, with HA subtypes H1, H3, H5, H6, H7 and H9 most commonly isolated.^38^, ^39^ In contrast, certain AIV lineages in subtypes H5 and H7 cause severe disease and rapid mortality in domestic birds and are referred to as highly pathogenic avian influenza (HPAI). While HPAI is phenotypically defined by a viruses lethality for susceptible chickens under strict experimental criteria,^40^ genetically, the key distinguishing structure of HPAI is the presence of a multibasic cleavage site (MBCS), which contains two to several basic residues (e.g. PQRE**RRRKR**/G) in the HA gene, as opposed to a single basic residue (PQRET**R/**G) in LPAI strains.^41^, ^42^, ^43^ Furin‐like peptidases, which are expressed ubiquitously in all tissue types of both avian and mammalian species, are responsible for the cleavage of HA at the MBCS site.^41^, ^44^ Additionally, HPAI strains have also emerged through non‐homologous recombination with a non‐HA protein nucleotide sequence, introducing a non‐native sequence into the HA gene.^45^, ^46^ Although this can enhance the pathogenicity of the virus, systemic replication does not necessarily occur in all species: experimental infection of ferrets and mice with HPAI H5N1 showed systemic virus replication,^47^, ^48^ but replication was only observed in the respiratory tract of non‐human primates,^49^ suggesting that pathogenicity of HPAI viruses is possibly host specific and multifactorial.^43^ It has been recently understood that domestic birds such as chicken lack the retinoic acid‐inducible gene‐I (RIG‐I), which is essential for signalling in the innate immune response,^50^, ^51^ as opposed to presence of RIG‐I in other avian species and mammals as an important sensor of viral RNA leading to activation of type I interferons.^52^ In experimental infection of chickens and ducks with H7N1 virus, increased viral pathogenesis with clinical signs of disease and death is observed in chickens, but infected ducks do not exhibit clinical signs, highlighting the evolutionary adaption of ducks being a natural host of the influenza virus.^53^, ^54^ There has also been back‐transmission of HPAI viruses from domestic poultry to wild bird populations that are implicated in bird die‐offs^55^ and intercontinental spread.^56^, ^57^, ^58^, ^59^

Most IAV spillover events to humans have occurred through close contact with domestic poultry.^2^, ^24^, ^60^ Poultry markets have been demonstrated to harbour a variety of influenza subtypes including H5N1, H6N1, H7N9 and H9N2; these viruses were detected from bird cages, drinking water troughs, environmental soils at live poultry markets and farms.^38^, ^61^, ^62^, ^63^, ^64^ Furthermore, live poultry markets act as a major source of reassortment of IAV causing the generation of new HA and neuraminidase (NA) subtype combinations and gene constellations, with the H9N2 subtype acting as a major donor of internal gene segments to other subtypes. Those viruses have subsequently caused human infection with H5N1, H5N6, H9N2, and H10N8 subtype viruses.^8^, ^38^, ^65^, ^66^, ^67^, ^68^, ^69^, ^70^, ^71^ The outbreak of H7N9 in China, a novel subtype that also contains H9N2 internal genes, has become one of the more severe zoonotic infections from avian IAV causing high morbidity and case fatality (40.6%) in humans.^72^, ^73^, ^74^ The majority of H7N9 human infection has resulted from close exposure to live poultry markets, whereas human‐to‐human transmission of this virus is limited.^74^ China is the world's largest producer of domestic poultry, with an estimated 13.1 billion poultry produced in 2015.^75^ These poultry include mainly chickens, ducks and geese with smaller numbers of a diverse range of species such as guinea fowl, partridge, pheasants and quails.^38^, ^76^ Production of domestic poultry ranges from high biosecurity commercial farms to backyard poly‐culture farming, where domestic poultry often come into contact with wild birds.^76^ Furthermore, the movement and mixing of domestic poultry to live poultry markets, where often multiple species are housed together regardless of origin, enhance the spread and mixing of IAVs.^76^, ^77^, ^78^, ^79^ Closure of wet markets in China has been shown to effectively reduce the transmission of H5N1, H9N2 and H7N9.^80^, ^81^, ^82^

## 4 Avian IAV adaptation to human and other mammalian hosts

In a large‐scale analysis of sequence data of IAVs from various hosts, several mutations were reported to be indicative of adaptation of IAVs from avian to other host species.^83^ However, in the surveillance of swine populations in southern China, many of these sites retain their avian‐like residues despite over 30 years of circulation in these mammalian hosts,^84^ suggesting these residues are likely founder effects rather than true adaptive mutations. Here, we highlight a few mutations that have been validated experimentally as critical in the adaptation of avian IAVs to humans and other mammalian hosts.

### 4.1 Avian HA vs mammalian HA

The HA protein is the key mediator of virus infection into the host cell and consists of two domains, HA1 and HA2. Three domains located in the HA1 globular head form the receptor binding site (RBS): the 130‐loop (residues 132–138), the 190 loop (residues 188–195) and 220‐loop (residues 221–228) (HA residues here follow H3 numbering unless otherwise stated).^32^, ^85^, ^86^, ^87^ Mutations within the RBS determine the specificity of binding to avian‐like α‐2,3‐sialic acid (SA) or mammalian‐like α‐2,6‐SA receptors. Specifically, the widening of the binding pocket through mutation favours α‐2,6‐SA receptor binding, as steric hindrance of the α‐2,6 *cis*‐linkage SA prevents binding to the narrower avian‐adapted RBS that binds to the smaller α‐2,3 *trans‐*linkage SA. Two mutations at Gln‐226‐Leu and Gly‐228‐Ser are required to widen the RBS in the human‐adapted H2 and H3 subtypes.^88^, ^89^, ^90^, ^91^ In comparison, the widening of the RBS in human‐adapted H1 subtype is determined by aspartic acid residues at positions 190 and 225, as they contribute to the conformational change of the 220 loop at the RBS in switching the binding affinity to the α‐2,6‐SA receptor.^92^, ^93^ In the case of subtype H5 virus, crystal structure studies show that the Gln‐226‐Leu mutation not only widens the binding pocket but also creates a hydrophobic environment, which alters the binding preference from avian α‐2,3 to the human α‐2,6 receptor.^94^, ^95^, ^96^, ^97^ Furthermore, mutations in the 130‐loop (Leu‐133‐Val/Ser and Ala‐138‐Val substitutions) of the HA‐H5 enable the virus to bind to both α‐2,3‐SA and α‐2,6‐SA receptors.^94^, ^98^, ^99^ Finally, mutations outside the RBS, specifically in glycosylation sites near the RBS, have also been determinants of receptor binding specificity, as these sites, when glycosylated, cause stearic hindrance to the optimal binding of respective SA linkage to the RBS, thereby restricting binding.^100^, ^101^, ^102^, ^103^, ^104^, ^105^

As mentioned earlier, the stability of the HA protein at various pH is associated with transmission in different hosts, with mammalian viruses having a lower optimum pH of conformational stability than avian viruses.^35^, ^106^, ^107^, ^108^ However, it was recently discovered that, in the adaptation from avian H1N1 to establishment of the European avian‐like (EA) swine virus lineage, this mammalian virus possessed a higher optimum pH of conformational stability than its avian counterpart, which is hypothesized to be mediated by changes in HA2 residues 72, 75, and 113.^109^ These findings certainly warrant the increased need to differentially characterize this trait between all susceptible species that can harbour IAVs for pandemic and panzootic preparedness as previously understood host‐specific optimum pH for HA stability may vary between subtypes and hosts.

### 4.2 Avian NA vs mammalian NA

The primary function of the NA protein is to cleave sialic acid receptors of host cells from where the virion has budded, thereby preventing reinfection into the same cell and promoting viral spread within the host.^7^ An optimal balance between the HA and NA protein function is required for effective infection and transmission of IAV: excess NA proteins can hinder the binding of HA to host cell receptors, whereas insufficient NA protein results in limited virus spread.^110^, ^111^ Avian NA proteins preferentially cleave α‐2,3‐SA residues, whereas mammalian NA proteins can cleave both α‐2,3‐SA and α‐2,6‐SA residues, indicating host‐specific adaptations of the protein.^112^ In avian IAV subtypes (e.g. H2N2, H7N1 and H9N2), deletions in the NA stalk are crucial for adaptation in chickens and other Galliformes poultry species.^113^, ^114^ While the significance of this deletion is yet to be elucidated, the naturally selected deletion serves as a barrier in interspecies transmission, as viruses containing the stalk region deletion were shown to have compromised transmission between ferrets.^115^

### 4.3 Avian PB2 vs mammalian PB2

The most widely cited mutation in polymerase PB2 subunit is Glu‐627‐Lys substitution, which is responsible for adaptation from avian to mammalian hosts. This mutation has been shown to mediate replication at a lower temperature (33°C) in the human upper respiratory tract, thereby facilitating efficient aerosol transmission, as opposed to a higher temperature (40°C) in avian intestinal tract.^116^, ^117^, ^118^ Additionally, this PB2 Glu‐627‐Lys mutation enhances the assembly of the viral ribonuclear protein (vRNP) complex in mammalian cells by stabilizing the binding of nucleoprotein (NP) and PB2 proteins during viral replication,^119^, ^120^, ^121^ and this stabilization further contributes in resistance to host RIG‐I sensing.^122^ Exceptions are found in H5N1 avian viruses as they are able to replicate in mammals successfully even with the absence of PB2 Glu‐627‐Lys mutation; however, a compensatory Asp‐701‐Asn mutation in the PB2 gene is present and has been found to facilitate adaptation to mammalian replication.^123^ This mutation promotes the transport of the RNA polymerase subunits into the nucleus through NP and PB2 interaction with importin‐α in mammalian cells, thereby promoting transmission in mammalian systems.^123^, ^124^

### 4.4 Avian NP vs mammalian NP

The NP segment plays a key role in interspecies transmission of IAV, particularly the switch from avian to mammalian hosts. The viral NP is associated with the sensitivity of the IAV to host myxovirus resistance A (MxA) proteins—an important intrinsic antiviral factor of mammals known to inhibit IAV as well as other RNA and DNA viral infections.^125^, ^126^, ^127^ It has been experimentally shown that an avian H5N1 virus with a reverse engineered human H1N1 NP protein can restrict the MxA sensitivity to eliminate species barrier and enhance viral replication 10‐fold,^125^, ^126^ indicating IAV can overcome the species barrier from an avian host to replicate successfully in the mammalian system through variation in the NP protein. For instance, the avian H7N9 virus has a crucial Asn52 mutation in the NP gene, causing a reduction in MxA sensitivity within human hosts.^128^ This also suggests that pre‐adaptive Asn‐52 mutation (i.e. resistance to MxA proteins) has been initiated in avian hosts prior to adaptation in mammalian hosts. This was demonstrated experimentally in squirrel monkeys, where the addition of an avian NP segment into a human H3N2 backbone attenuated virus growth.^129^, ^130^, ^131^

### 4.5 Avian NS vs mammalian NS

The non‐structural protein 1 (NS1) protein has been implicated in variable virulence and pathogenicity within different hosts through its interactions of PDZ‐binding motif at the C terminus of the protein.^83^, ^132^, ^133^, ^134^ In a large‐scale comparative genomic approach of all IAV, a specific 11 amino acid long sequence feature variant type (SFVT) located between residues 137–147 of the NS1 protein was statistically associated with a host restriction phenotype,^135^ although this has yet to be tested experimentally. Nonetheless, discovery of this SFVT emphasizes the importance of further studying this protein and its potential role in interspecies species transmission.

Mutations in the non‐structural protein 2 (NS2)/nuclear export protein (NEP) gene has of late received more attention as an important factor in mammalian adaptation of avian IAV, due to its role as a regulator of transcription and replication of influenza virus genome within the host cell.^136^, ^137^, ^138^ Importantly, it was shown that substitution Met‐16‐Ile in the NEP conferred H5N1 avian polymerases the ability to efficiently replicate in mammalian cells by stabilizing the vRNP complex and by enhancing vRNA synthesis.^138^

### 4.6 Avian PB1‐F2 vs mammalian PB1‐F2

The complete role of PB1‐F2 in the virus lifecycle still remains to be elucidated, as it has been shown to both aid and disrupt viral virulence. It is generally understood that PB1‐F2's role is subtype and host dependent. It has been demonstrated that within the 1918‐like H1N1 viruses, PB1‐F2 interacts with the PB1 protein to increase replication by retaining the polymerase complex in the nucleus longer.^139^ In HPAI H5N1, the wild‐type PB1‐F2 protein was found to have no effect on the severity of infection in mice beyond replication in the lung, but replication was detected systemically in multiple organs of experimentally infected ducks, further supporting the existence of a host‐specific function for the protein.^140^, ^141^

### 4.7 Avian CpG vs mammalian CpG non‐coding region

Mutations in the non‐coding region of IAV have been shown to mediate cross‐species transmission. These non‐coding adaptations are due in part to constraints on host mechanisms that restrict the probability of mutational fixation, and consequently, a non‐random pattern of mutation is seen across the virus genome in different host species.^142^ For instance, many RNA viruses evolve to mimic the CpG dinucleotide frequencies of their hosts.^143^, ^144^ Mammalian DNA sequences contain unmethylated CpG motifs responsible for inducing innate immune responses through interactions with Toll‐like receptor 9 (TLR9)^144^, ^145^; however, most mammals protect themselves against autoimmunity by maintaining CpG dinucleotides that are methylated and at low frequency.^145^ To accommodate this, IAV has been observed to evolve lower dinucleotide CpG content following interspecies transmission.^143^, ^146^ This phenomenon has also been experimentally confirmed, where the infection with modified human A/WSN/33 influenza virions expressing avian‐like increased CpG content was shown to increase ssRNA recognition and immune activation by human plasmacytic dendritic cells.^144^ This low CpG preference has been reported in human IAV subtypes such as the H1N1 and H3N2 in comparison with avian IAVs.^143^ However, this trend was not observed in H2N2 isolates from the 1957 pandemic, likely due to the short duration in which this subtype circulated in humans.^147^

Another parameter of codon usage bias is relative synonymous codon usage (RSCU), which is commonly applied to IAV genomes as a metric of comparison between different host species.^142^ A clear distinction in codon usage patterns has been shown in H1N1 and H3N2 subtypes by comparison among different hosts: avian‐derived human and swine viruses demonstrate RSCU values that become closer to their new mammalian hosts over time, and do not show preference for the synonymous codons used in their precursor avian hosts.^142^, ^148^, ^149^, ^150^ Other IAV subtypes (including H2N2, H5N1 and H9N2) that have transmitted to humans have RSCU values that are similar to those of avian‐like RSCU, as these subtypes have been transmitted directly from an avian source and have not successfully circulated long enough in humans to establish an optimized codon usage in mammals.^142^, ^148^, ^151^

## 5 Pigs: the mixing vessel?

Pigs are widely recognized as a “mixing vessel” of IAV with the presence of both α‐2,3‐SA and α‐2,6‐SA residues distributed throughout their respiratory tracts, where avian, swine and human IAV strains reassort following co‐infection and give rise to new genetic variants, potentially leading to epidemics and/or epizootics.^152^ The most remarkable example is the swine‐origin 2009 H1N1 pandemic (H1N1/2009), which emerged from the reassortment of gene segments from the H1N1 European “avian‐like” swine (EA‐swine) lineage, H1N2 triple reassortant swine (TRS) lineage and an Eurasian avian H1N1 lineage.^153^ Several adaptive changes of specific host residues have been reported that contributed to the establishment of this swine virus in humans, observed especially from viruses isolated during the early phase of the pandemic, with the later circulating viruses showing mutations involved in antigenic escape within human hosts.^154^ The 2009 pandemic H1N1 (pH1N1) viruses did not contain the Lys‐627 or Asn‐701 substitutions that were considered indicative of mammalian adaptation,^43^, ^155^, ^156^ and it now appears these substitutions are not required for swine to human transmission. Indeed, when the mammalian‐like Lys‐627 and Asn‐701 residues were introduced, a significant increase in polymerase activity and viral pathogenesis was observed,^155^, ^156^ but this replicative advantage of the mutation was not necessary for the initial establishment of the avian polymerase in TRS lineage and subsequently human pH1N1 viruses.^157^ Instead, partial compensatory mutations Gly590Ser and Glu591Arg were observed to aid polymerase activity of in the lack of the mammalian 627 and 701 residues, providing a possible explanation for the successful transmission of the avian‐like residues in mammals.^43^, ^155^, ^156^, ^157^ Additionally, the H1N1/2009 virus has reduced sensitivity to host MxA proteins though the interaction with the NP gene, as a result of the TRS‐origin NP reassortment.^120^, ^121^, ^158^

There have been several spillover infections of swine IAVs into humans^159^, ^160^, ^161^; however, increasing evidence suggests reintroduction of the human virus into swine populations is more common.^162^, ^163^ The susceptibility of swine infection by human virus can be attributed to, among many options, the dynamics of host immune development and farming practices. The immune response to IAV infection in swine is similar to that of the human antiviral responses, with experimental evidence showing similar activation and secretion of cytokines is triggered in response to infection with the same virus.^164^, ^165^ Unlike humans, swine are unable to transfer maternal immunoglobulins to porcine embryos due to the existence of a thick placenta comprising six tightly connected tissue layers.^166^ The first 24 hours after birth of the newborn pigs is exceptionally crucial for the transfer and intestinal absorption of maternal IgG from colostrum.^166^, ^167^, ^168^ Although maternal antibodies through suckling can protect newborn piglets to some extent, the piglets are still highly susceptible to new infections. High‐density commercial farming practices, where piglets are typically sent to market at 6 months of age, can therefore have profound effects on the transmission and diversity of IAVs in various swine populations.^162^, ^163^

## 6 Equine IAV

Two equine IAV subtypes, H7N7 and H3N8, were first detected in the 1950s, although the host origin of the former subtype is not known.^169^ However, as the H7N7 equine lineage has not been detected in over three decades, it is possible the H7N7 has become extinct in equine hosts.^170^, ^171^, ^172^ Despite its disappearance, the lineage has been studied extensively to elucidate the factors involved in its emergence and potential virulence in mammalian species.^173^, ^174^, ^175^ Of note, it has been observed that the equine H7N7 contains an MBCS in the H7 HA protein,^176^ which in avian H7 lineages have been implicated in HPAI infections in poultry and have infected humans.^177^ The equine lineage virus was also found to be highly pathogenic and neurovirulent in mice without prior adaptation.^173^ However, the intracellular cleavage of the equine H7N7 lineage was found to be due to an 11 amino acid motif adjacent to the MBCS that, when inserted into an LPAI H7N3 virus, increased the pathogenicity of infection.^175^

In contrast, the H3N8 equine lineage may have originated wholly from an avian IAV source, and these viruses have been shown to undergo frequent intersubtype and intrasubtype reassortments.^178^, ^179^, ^180^ Interestingly, the H3N8 virus in equine hosts preferentially binds to avian‐like α‐2,3‐SA, where the receptors are abundantly present in the upper respiratory tract of horses.^181^, ^182^ As such, horses are always considered as dead‐end hosts, but the interspecies transmission of H3N8 virus from horses to dogs and camels raises the question of whether horses can act as mixing vessels for IAV.^169^, ^182^

## 7 Canine IAV

IAV has been detected in canine species since the early 2000s, and there are currently two stably transmitting lineages of IAVs that circulate in dogs: the equine‐derived H3N8 and the avian‐derived H3N2 strains.^169^ The transmission of the equine H3N8 subtype to dogs seems to have occurred *en bloc* through close contact with infected horses^183^ and this virus continues to circulate in dogs with no evidence of reassortment with other subtypes to date.^169^ This is further supported by recent findings that despite phylogenetic divergence and genetic change from the equine lineage, canine H3N8 was not observed to be phenotypically different from equine H3N8 strains.^184^ The avian‐derived H3N2 canine IAV has a much broader host range, and unlike the H3N8 lineage, reassortment with other subtypes, such as with H5N1 and H1N1, has been detected.^185^, ^186^ Recently, an isolated case of an H6N1 virus was detected a dog in Taiwan, likely through contact with infected chicken, and represents the potential for other avian IAV subtypes to emerge in canid hosts.^187^ Lineage‐specific mutations have been detected in the canine IAVs that suggest adaptive mutations in various segments towards the new canine hosts,^188^, ^189^ but only the role of a leucine residue at position 222 (H3 numbering) has been confirmed as a host‐specific adaptation thus far.^190^, ^191^

## 8 Concluding thoughts

There is a need to understand adaptations in IAV that confer interspecies transmission capabilities to drive active surveillance for these mutations in nature and prevent an impending outbreak. Thus, the continued evolution of influenza viruses in its myriad of hosts necessitates further studies that characterize the risk of human transmission in these viruses, to provide a meaningful assessment of pandemic potential. Furthermore, the benefits of such efforts have been seen in the identification and rapid response to recent H7N9 outbreaks in China,^12^, ^73^ while other studies that have characterized mutations that may enable sustained human‐to‐human transmission or describe pathogenesis have been useful for risk assessment of contemporarily circulating IAVs.^104^, ^107^, ^108^ However, as there is continued mixing of viruses normally resident in different species, there is value in the continued experimental characterization of circulating viruses, particularly from subtypes known to have caused pandemics and those viruses present in animals to which humans have high exposure.
